# Determinants of Learners’ Continuance Intention in Informal Video Learning: Evidence from YouTube

**DOI:** 10.3390/bs16091574

**Published:** 2026-09-04

**Authors:** Tewarit Khanmolee, Somchai Lekcharoen, Sumaman Pankham

**Affiliations:** College of Digital Innovation Technology, Rangsit University, Pathum Thani 12000, Thailand; tewarit.k67@rsu.ac.th (T.K.); sumaman.p@rsu.ac.th (S.P.)

**Keywords:** continuance intention, informal learning, YouTube, structural equation modeling, satisfaction, expectation confirmation model, necessary condition analysis

## Abstract

Informal video platforms support self-directed learning, yet the determinants of learners’ continuance intention to use YouTube for informal learning remain insufficiently integrated. This study addressed two questions: how the hypothesized structural relationships in an extended expectation–confirmation model explain Thai learners’ continuance intention and which determinants are necessary for high continuance intention. A sequential exploratory mixed-methods design was employed. Fuzzy e-Delphi with 23 experts retained 33 measurement items; survey data from 797 Thai learners were analyzed using covariance-based structural equation modeling, bootstrapped specific indirect effects, and Necessary Condition Analysis (NCA). The model explained 74% of the variance in continuance intention. Satisfaction was the strongest direct predictor (β = 0.559), followed by perceived enjoyment and trust. All 12 hypothesized structural relationships and eight indirect effects were statistically significant, revealing interconnected roles for confirmation, content quality, subjective norm, perceived enjoyment, perceived usefulness, satisfaction, and trust. NCA identified satisfaction and content quality as the principal necessary conditions, with smaller supporting roles for perceived usefulness and perceived enjoyment. Theoretically, the findings extend the expectation–confirmation model to voluntary informal video learning and distinguish contributing relationships from necessary constraints. Practically, they support prioritizing high-quality content and satisfactory learning experiences while strengthening perceived usefulness and perceived enjoyment.

## 1. Introduction

The rapid advancement of information and communication technologies, combined with the global disruption caused by the pandemic, has accelerated the transition toward digital learning environments, enabling flexible and accessible learning beyond traditional classroom settings (Gao & Liu, 2023; Sumi, 2024). Among various digital platforms, online video sharing platforms such as YouTube have evolved from primarily entertainment-oriented media into widely recognized resources for informal learning. Prior research indicates that YouTube provides extensive access to diverse, user-generated content, facilitating flexible, cost-effective, and self-directed learning opportunities for global users (Quintero-Rodríguez & Colás-Bravo, 2024; Habibi et al., 2024).

YouTube has become one of the most widely used platforms globally, with over two billion users and more than one billion hours of video content consumed daily (Cho et al., 2023; Gao & Liu, 2023). Informal learning can be viewed as a self-directed process in which individuals develop knowledge outside formal educational institutions, with learners deciding what, when, and how they learn (Quintero-Rodríguez & Colás-Bravo, 2024). The long-term success of digital learning systems largely depends on users’ continuance intention, which reflects sustained engagement over time (Ashrafi et al., 2020).

Within technology acceptance research, the technology acceptance model (TAM) explains why users accept a system through two key beliefs: perceived usefulness (PU) and perceived ease of use (Sumi, 2024; AL-Hawamleh, 2024). However, the TAM primarily focuses on pre-adoption behavior and provides limited insight into users’ post-adoption decisions. In contrast, the expectation confirmation model (ECM) offers a more suitable framework for understanding continuance intention by highlighting the role of confirmation (CON) and satisfaction (SAT) in shaping users’ continued usage behavior (Inan et al., 2023; Sukma Ningsih et al., 2024). Prior studies in various e-learning contexts have also identified that online learning environments often face challenges related to learner discontinuance and low retention, particularly in open and self-directed learning settings (Huang & Liu, 2024; Rekha et al., 2023).

Emerging research suggests that continuance intention in digital learning environments is influenced by a broader set of cognitive, affective, and social factors. For example, perceived enjoyment has been identified as an intrinsic motivational factor associated with continued engagement in online learning (Huang & Liu, 2024; Habibi et al., 2024). In addition, content quality has been shown to influence users’ perceptions and SAT in e-learning systems (AL-Hawamleh, 2024; Cho et al., 2023). Social influence, particularly subjective norms, has also been incorporated into extended technology acceptance frameworks to explain user behavior (Ashrafi et al., 2020; Habibi et al., 2024). Furthermore, trust has been found to play a meaningful role in shaping users’ perceptions and their intention to continue using video-based platforms (Xuan et al., 2024).

Despite these advancements, previous studies have generally examined cognitive, affective, content-related, social, and trust-related determinants separately or within different theoretical models. Consequently, their joint direct and indirect relationships remain insufficiently integrated, particularly in voluntary, YouTube-based informal learning. Moreover, empirical evidence from developing and Southeast Asian contexts remains limited. Thailand therefore provides a relevant national context for examining continuance intention in informal YouTube learning, although findings from this setting require validation in other countries and cultural contexts.

Most continuance-intention studies rely on sufficiency-based methods, such as regression and SEM. These methods are useful for estimating the net associations among variables specified in a proposed model, but they do not directly determine whether a condition must reach a minimum level for a high level of continuance intention to occur. This distinction is relevant to YouTube-based learning because a favorable level of one factor may not compensate for an insufficient level of another. NCA identifies conditions that are necessary but not sufficient, for achieving an outcome (Dul, 2016); its interpretation should consider effect sizes, statistical significance, and bottleneck levels (Dul et al., 2020, 2023). The method has also been applied in educational research (Tynan et al., 2020). Combining SEM and NCA offers a more diagnostic view of continuance intention: SEM estimates net structural relationships, whereas NCA identifies empirically necessary conditions and bottlenecks without implying causality.

In response to these gaps, this study develops and tests an extended ECM integrating content quality, subjective norm, perceived enjoyment, and trust to explain Thai learners’ continuance intention to use YouTube for informal learning from complementary sufficiency and necessity perspectives. Accordingly, the study addresses the following research questions: 

RQ1: How do the hypothesized structural relationships in the proposed extended ECM explain Thai learners’ continuance intention to use YouTube for informal learning?

RQ2: Which determinants in the model constitute necessary conditions for achieving a high level of Thai learners’ continuance intention to use YouTube for informal learning?

This study makes three contributions. First, it extends the ECM to voluntary YouTube-based informal learning by incorporating content quality, subjective norm, perceived enjoyment, and trust alongside confirmation, perceived usefulness, and satisfaction. Second, the combined use of CB-SEM and NCA distinguishes the structural relationships among the constructs from the conditions and bottlenecks necessary for achieving high continuance intention. Third, the findings offer educators, content creators, and platform designers an empirical basis for deciding which aspects of the learning experience should receive priority when seeking to strengthen learners’ intention to continue using YouTube for informal learning.

## 2. Literature Review

### 2.1. Theoretical Foundation

Understanding why learners keep coming back to a platform is different from explaining why they first used it. The TAM mainly accounts for early-stage technology acceptance, where perceived usefulness and ease of use shape a person’s decision to try a system (Davis, 1989). The ECM offers a useful basis for explaining this post-adoption stage (Bhattacherjee, 2001). The ECM shifts the focus to how confirmation of expectations and subsequent SAT drive a user’s decision to continue. Yet, the ECM alone does not capture the full picture of informal learning. YouTube is not a purely cognitive environment; it involves content-related judgments, emotional responses, social dynamics, and issues of trust. To account for these dimensions, recent studies have increasingly extended the ECM by integrating complementary theoretical perspectives. For instance, Huang and Liu (2024) incorporated perceived enjoyment from Self-Determination Theory (Deci & Ryan, 1985) into the ECM framework, demonstrating that intrinsic pleasure plays a strong mediating role in asynchronous online learning. Similarly, D. M. Nguyen et al. (2021) extended the ECM based on DeLone and McLean’s IS success model (DeLone & McLean, 2003) and trust theory (Gefen et al., 2003) to explain continuance intention toward digital services. Beyond ECM-based extensions, other studies highlight the unique characteristics of modern digital platforms. Cho et al. (2023) examined content quality as a critical driver of user satisfaction and continuance intention specifically on YouTube, emphasizing the platform’s open content nature. Meanwhile, Rahmayanti et al. (2022) demonstrated the significant roles of subjective norm drawn from the Theory of Planned Behavior (Ajzen, 1991) and trust in shaping continuance intention among younger generations. When viewed as a whole, these studies indicate that relying on only one theory may not fully explain learners’ continued use of YouTube. Therefore, the present research develops a model that links the ECM with content quality, subjective norm, perceived enjoyment, and trust to explain learners’ continuance intention on YouTube. Accordingly, the following subsections organize the literature around the relationships among the constructs and integrate the relevant theoretical reasoning, empirical evidence, and hypotheses.

### 2.2. Literature Review and Hypothesis Development

#### 2.2.1. Relationships of Confirmation with Perceived Enjoyment and Satisfaction

Confirmation (CON) entails assessing whether actual experience matches pre-usage expectations, serving as the core of the ECM (Bhattacherjee, 2001). PE refers to the pleasure experienced during technology use (Davis et al., 1992). Empirical evidence clearly indicates that CON positively influences PE across e-learning systems and online libraries (Ashrafi et al., 2020; L. Li et al., 2022; Ramadhan et al., 2022). Studies across asynchronous learning and online collaborative environments similarly report a positive relationship between confirmation and perceived enjoyment (Huang & Liu, 2024; Inan et al., 2023). Fulfilling expectations in MOOCs also acts as a strong antecedent for elevating intrinsic motivation and enjoyment (Rekha et al., 2023). However, prior research tested this influence primarily in formal learning environments, such as university LMS (Ashrafi et al., 2020), MOOC structures (Rekha et al., 2023), online libraries (Ramadhan et al., 2022), or mandatory e-learning (L. Li et al., 2022). The relational mechanisms in informal learning contexts remain unclear. Highly autonomous environments intricately blending utilitarian and hedonic content may produce an enjoyment generation mechanism entirely different from that of traditional educational systems. This leads to the following hypothesis:

**H1.**  *Confirmation positively influences perceived enjoyment.*

Within the ECM, confirmation should increase satisfaction because users evaluate an experience more favorably when its performance meets or exceeds their prior expectations (Ashrafi et al., 2020; Sumi, 2024). Sukma Ningsih et al. (2024) state that students are highly satisfied and driven to continue using YouTube when it provides a better experience than expected. This concept is broadly supported across e-learning contexts (Ashrafi et al., 2020; Du et al., 2024; Meng & Li, 2024; Rahimullah et al., 2022; Sumi, 2024) and other social media platforms (Jia et al., 2023; Khan & Saleh, 2023; Tsai et al., 2020). However, L. Li et al. (2022) found that CON has no direct effect on satisfaction in online learning, acting only indirectly via perceived usefulness and enjoyment. This indicates that, in informal learning, merely meeting expectations may not be enough to directly trigger satisfaction. Accordingly, we hypothesized that

**H2.**  *Confirmation positively influences satisfaction.*

#### 2.2.2. Relationships Between Content Quality and Satisfaction, Perceived Usefulness, and Trust

Content quality (CQ) refers to a broad information-related construct commonly assessed through accuracy, relevance, timeliness, and information adequacy, although its operationalization varies across studies (Cho et al., 2023; Khan & Saleh, 2023; Rahimullah et al., 2022; Saggu & Mohan, 2025; Sumi, 2024). For instance, Cho et al. (2023) evaluated YouTube CQ through relevance, timeliness, informativeness, and sufficiency, whereas Sumi (2024) emphasized accuracy, validity, and availability. Despite these contextual variations, there is a strong consensus that high-quality content is a fundamental determinant of the success of digital platforms and online learning systems (Lashayo & Mhina, 2022; Legramante et al., 2023; Pang et al., 2020; Piaralal et al., 2023). Extensive empirical evidence demonstrates that information quality significantly drives user satisfaction (Lashayo & Mhina, 2022; Legramante et al., 2023; Pang et al., 2020; Rahimullah et al., 2022). For example, Cho et al. (2023) highlighted the critical role of CQ in sustaining YouTube viewer satisfaction. Similar positive relationships have been established across various digital contexts: Saggu and Mohan (2025) found a strong positive effect in over-the-top (OTT) related video service context; Khan and Saleh (2023) identified information quality as the strongest predictor of satisfaction among Facebook users; and Lee and Sung (2023) confirmed a significant positive link between the CON of information quality and user satisfaction in cryptocurrency exchanges. However, this relationship is not universally consistent. In contrast to the aforementioned studies, Sumi (2024) reported no statistically significant link between information quality and user satisfaction within an e-learning environment in a developing country. This anomaly suggests that, in certain contexts, users may prioritize other systemic factors or service dimensions over the content itself. Given these mixed empirical results, the precise mechanisms through which CQ influences satisfaction require further contextual investigation. The hypothesis is as follows:

**H3.**  *Content quality positively influences satisfaction.*

CQ refers to the comprehensibility and relevance of the provided material (DeLone & McLean, 2003). Conversely, PU is learners’ belief that using a particular system can improve their learning performance (Davis, 1989). High-quality content is widely recognized as a fundamental pillar of effective digital learning systems (Al-Fraihat et al., 2020). Several recent studies report that content quality exerts a positive influence on PU. For instance, Lee and Sung (2023) demonstrated that relevant and accurate content significantly elevates learners’ perceived usefulness, a finding consistently corroborated by multiple recent studies (AL-Hawamleh, 2024; Alkhuwaylidee, 2025; Dwi Lestari & Riatun, 2024; Suzianti & Paramadini, 2021). Notably, Abu-Taieh et al. (2022) found a strong positive effect of information quality on information usefulness specifically in the context of YouTube as a learning tool (β = 0.861), providing direct evidence within the same platform examined in this study. However, the assumption that superior content inherently translates to perceived usefulness is not universally supported. Revealing highly conflicting outcomes, Legramante et al. (2023) found that information quality had no significant direct effect on PU. This discrepancy aligns with an emerging stream of research suggesting that high-quality content does not automatically equate to perceived usefulness from the students’ perspective (Alshammari & Alshammari, 2024; Lashayo & Mhina, 2022; Zheng et al., 2023). In light of these insights, the following hypothesis was established.

**H4.**  *Content quality positively influences perceived usefulness.*

CQ is vital for establishing user reliance in digital environments. For instance, Almuqrin et al. (2022) conceptualized data quality in terms of accuracy, truthfulness, completeness, and up-to-dateness, positing that such quality fundamentally mitigates user uncertainty and fosters a psychological state of trust. Empirical evidence broadly confirms a positive relationship between content quality and trust. High-quality information significantly enhances user trust across various platforms, including e-commerce, e-government, social commerce, and formal online learning (Hooda et al., 2023; Khwaja et al., 2020; H. Li et al., 2025; Truc & Tuan, 2020). When users perceive that a system provides accurate and useful information, their inclination to trust the platform strengthens (Khwaja et al., 2020). Specifically within the context of formal online learning, H. Li et al. (2025) found that information quality directly influences trust, which then acts as a mediator toward students’ continuance intention. Despite its importance, the effect of CQ on trust is not universally absolute. Purwanto et al. (2020) reported a contrasting result, showing that users’ perceived data quality was not a significant predictor of trust. Instead, they found that perceived service quality played a stronger role in shaping citizens’ trust in open government data. Building on these contextual gaps and mixed findings, the following hypothesis was formulated.

**H5.**  *Content quality positively influences trust.*

#### 2.2.3. Relationships Between Subjective Norm and Trust

Subjective norm (SN) represents the social influence that individuals perceive from important people around them, including family, friends, or peers, when making a behavioral decision (Barattucci et al., 2022; Fauzi et al., 2023; Solomon, 2021). The need for social approval acts as a basic motivation that can shape trust when people utilize new systems or services (Cicin & Çetin Gürkan, 2026; Rahmayanti et al., 2022). Several SEM-based studies point out a positive relationship between subjective norms and trust across diverse sectors. Solomon (2021) confirmed this dynamic in internet banking. In studies of AI adoption, Cicin and Çetin Gürkan (2026) demonstrated that subjective norms significantly enhance physicians’ trust in AI technologies, which fully explains the link with their intention to use these systems; Rahmayanti et al. (2022) observed that SN significantly influence trust among both Millennials and Zillennials. In the context of healthcare technology, W. Li et al. (2024), revealed that subjective norms significantly and positively influence older adults’ trust in telemedicine services. Moreover, in public health, Barattucci et al. (2022) found that perceived social consensus acts as a full mediator; SN builds trust in science, which then drives the intention to get vaccinated. Fauzi et al. (2023) demonstrated that SN lacks a significant direct effect on trust regarding private health insurance, likely due to conflicting societal values. This finding stands in contrast to the majority of studies that support a positive link. Given these mixed and context-dependent findings, the relationship between subjective norm and trust requires further examination in an informal and voluntary learning environment such as YouTube. Therefore, the following hypothesis was proposed:

**H6.**  *Subjective norm positively influences trust.*

#### 2.2.4. Association of Perceived Enjoyment and Perceived Usefulness with Satisfaction and Continuance Intention

Perceived enjoyment (PE) captures the pleasure users experience during system use (Du et al., 2024; L. Li et al., 2022; Ramadhan et al., 2022). In this study, PE is treated as an activity-specific affective belief that can support intrinsic motivation but remains conceptually distinct from the broader motivational construct. It shapes how users react emotionally and builds the foundation for their overall satisfaction (Pereira & Tam, 2021). Extensive studies consistently confirm that PE significantly enhances user satisfaction across diverse digital platforms (Deng et al., 2023; Du et al., 2024; Inan et al., 2023; Kim & Chung, 2023; Ramadhan et al., 2022). Specifically, Gil-Cordero et al. (2023) highlighted that PE is closely linked to learners’ satisfaction when using tools like YouTube and TikTok. L. Li et al. (2022) and Pereira and Tam (2021) also demonstrated that enjoyment acts as a primary catalyst driving satisfaction in online learning and video-on-demand environments. However, the assumption that enjoyment universally translates into satisfaction presents mixed results in certain digital learning contexts. Rekha et al. (2023) discovered that PE did not significantly influence user satisfaction in MOOCs. Similarly, J. Li et al. (2025) reported unexpected results in smart learning platforms, where perceived enjoyment did not significantly improve satisfaction and showed a negative, non-significant direction. Thus, the following hypothesis was proposed:

**H7.**  *Perceived enjoyment positively influences satisfaction.*

PE describes the extent to which learners find a system enjoyable during use and can function as an activity-specific source of intrinsic motivation in technology use research (Davis et al., 1992). In post-adoption learning contexts, prior studies suggest that enjoyment plays an important role in supporting learners’ continued use of digital platforms. For example, PE has been found to significantly predict continuance intention in the use of online library resources (Ramadhan et al., 2022) and in students’ continued use of English learning applications in non-mandatory environments (Du et al., 2024). More recent evidence further indicates that PE can serve as a strong predictor of continuance intention (H. Li et al., 2025) while also exerting indirect effects through mediating mechanisms such as perceived usefulness. Notably, PE has been shown to influence continuance intention both directly and indirectly, indicating a partial mediation structure (Huang & Liu, 2024). Related studies in e-learning and hybrid learning contexts also support its positive role, although the magnitude of its effect may vary across settings (Wang et al., 2026; Inan et al., 2023; Ashrafi et al., 2020). However, existing research has predominantly focused on structured educational environments, while less is known about how enjoyment shapes continuance intention in highly autonomous informal learning environments such as YouTube. Accordingly, the following hypothesis is presented:

**H8.**  *Perceived enjoyment positively influences continuance intention.*

Drawing on foundational technology acceptance research, perceived usefulness refers to users’ belief that a system can help them complete tasks more effectively (Davis, 1989, as cited in Zhou et al., 2022). PE, on the other hand, captures the intrinsic pleasure derived from using the system itself (Venkatesh, 2000, as cited in Zhou et al., 2022). In educational technology contexts, the interaction between affective enjoyment (PE) and utilitarian value (PU) constitutes a key mechanism for predicting learners’ behavioral intentions. A growing body of empirical evidence suggests that PE positively predicts PU across a range of technology-mediated learning environments. When learners experience enjoyment while interacting with a system, they tend to judge the system as more useful and practically helpful. For example, Humida et al. (2022) reported a significant positive influence of PE on PU in university e-learning contexts. Similarly, Koutromanos et al. (2024) identified this relationship in the adoption of Mobile Augmented Reality technologies. Fan and Wang (2023) and Huang and Liu (2024) further support this linkage, although the latter situates the relationship within a serial mediation framework involving CON, PE, and PU. However, the role of PE in shaping PU within fully informal learning environments remains less clearly understood. Previous studies have mainly examined structured, instructor-regulated settings (Fan & Wang, 2023). Interestingly, evidence from outside education suggests a more complex dynamic. Holdack et al. (2022), in a retail AR context, found that PE can exert a stronger direct influence on user attitudes than PU, challenging the traditional emphasis on utilitarian value in acceptance models. Accordingly, the following hypothesis was formulated:

**H9.**  *Perceived enjoyment positively influences perceived usefulness.*

Perceived usefulness (PU) captures users’ belief that a platform can improve their performance (Davis, 1989). The relationship between PU and SAT constitutes a key explanatory mechanism in information systems research, as it captures how users translate cognitive evaluations of usefulness into affective responses that influence post-adoption behavior (Bhattacherjee, 2001; Pan et al., 2024). A substantial body of empirical research supports a positive relationship between perceived usefulness and satisfaction across digital learning environments. For instance, L. Li et al. (2022) found that PU significantly enhanced user satisfaction in online learning during the COVID-19 pandemic. Similarly, Legramante et al. (2023) identified PU as the strongest predictor of satisfaction in Moodle-based learning systems, while Pan et al. (2024) confirmed its significant positive effect among adult learners in online education platforms. Consistent findings have also been reported in diverse contexts, including primary education (Suzianti & Paramadini, 2021), MOOCs (Rekha et al., 2023), and informal online learning environments (Meng & Li, 2024). In addition, Ramadhan et al. (2022) demonstrated that PU significantly increases satisfaction, although its influence on continuance intention operates indirectly through satisfaction. Collectively, these studies suggest that, when learners perceive clear academic benefits from digital platforms, their SAT tends to increase (Soria-Barreto et al., 2021). However, despite this general consensus, recent empirical evidence reveals important inconsistencies in the PU-satisfaction relationship. For example, J. Li et al. (2025) found that PU does not significantly influence SAT in smart learning platforms within formal institutional contexts, suggesting that functional performance may be perceived as a baseline expectation rather than a source of affective evaluation. Ashrafi et al. (2020) and Al-Emran et al. (2020) likewise reported that PU significantly enhances satisfaction. Taken together, these mixed results suggest that the influence of PU on SAT may vary depending on contextual factors such as learning autonomy, platform type, and user expectations. In particular, the dynamics of this relationship remain underexplored in informal, self-directed learning environments such as YouTube, where users are not constrained by institutional requirements. The following hypothesis was developed:

**H10.**  *Perceived usefulness positively influences satisfaction.*

#### 2.2.5. Relationships Between Satisfaction and Trust and Continuance Intention

Satisfaction (SAT) has been widely recognized as a central construct in explaining users’ continuance intention in technology-mediated learning environments. Rooted in the expectation confirmation framework, SAT reflects users’ post-use evaluation formed through how users judge their earlier expectations against what they actually experience. A substantial body of empirical research consistently demonstrates that satisfaction is positively associated with users’ intention to continue using digital learning systems in emergency online learning during the COVID-19 period, and in blended learning, MOOCs, and video-based learning platforms (Jang & Chiang, 2024; L. Li et al., 2022; Shang & Lyv, 2022; Yang et al., 2023; Pan et al., 2024; Zhao et al., 2025). For example, Sukma Ningsih et al. (2024) found that students’ SAT significantly motivated their intention to keep using YouTube for study purposes after the post-pandemic context. Similarly, Cho et al. (2023) confirmed that user satisfaction significantly drives continuous use intention specifically on YouTube (β = 0.255), reinforcing the applicability of this mechanism within the same platform context. However, emerging evidence suggests that the role of SAT may vary depending on contextual and psychological mechanisms. Recent studies have further highlighted that SAT may operate within a broader affective process rather than functioning as a standalone predictor. Xuan et al. (2024), for example, demonstrated that satisfaction not only directly influences continuance intention but also exerts an indirect effect through trust, which serves as a key mediating mechanism linking affective responses to behavioral outcomes. Similarly, Jia et al. (2023) argued that perceived value, conceptualized as a trade-off between perceived benefits and sacrifices, can complement satisfaction and enhance the explanatory power of continuance intention models. Taken together, these findings indicate that, while SAT remains a fundamental determinant of continuance intention, its effect may be contingent upon additional mediating and contextual factors, particularly in dynamic and informal learning environments such as YouTube. Consistent with the above analytical perspective, the following hypothesis was proposed:

**H11.**  *Satisfaction positively influences continuance intention.*

Trust (TRU) is widely acknowledged as a central sociopsychological mechanism in post-adoption contexts, particularly in digital environments characterized by uncertainty and perceived risk. By reducing users’ concerns regarding security, reliability, and system performance, trust facilitates users’ willingness to sustain long-term interactions with digital platforms. Substantial empirical evidence consistently supports the positive role of trust in predicting continuance intention across multiple domains. For instance, trust significantly enhances continuance intention in FinTech peer-to-peer payment systems (Savitha et al., 2022), mobile payment services (Kaewkitipong et al., 2022), and health science video platforms (Xuan et al., 2024). In the context of AI-enabled services, D. M. Nguyen et al. (2021) further demonstrated that trust is the strongest direct predictor of continuance intention toward banking chatbot services, exceeding the effects of both SAT and PU. Similarly, in online learning environments, TRU has been shown to operate as a key mediating mechanism through which platform characteristics influence users’ continuance intention (H. Li et al., 2025). However, despite this general consensus, emerging evidence reveals that the effect of trust is not universally stable and may depend on contextual conditions. In mobile shopping, for example, trust is not directly linked to continuance intention at a statistically significant level but instead influences it indirectly through user adaptation (G.-D. Nguyen & Ha, 2021). Moreover, in institutionally mandated learning environments, TRU may even produce unintended outcomes; specifically, it has been found to negatively affect user satisfaction, suggesting a potential mismatch between institutional trust and users’ experiential expectations J. Li et al. (2025). These inconsistencies indicate that the role of trust is highly context dependent and may vary according to levels of user autonomy, platform type, and usage conditions. Accordingly, the function of perceived trust in informal, self-directed learning environments such as YouTube remains insufficiently understood. Consistent with the above analytical perspective, the following hypothesis was proposed:

**H12.**  *Trust positively influences continuance intention.*

### 2.3. Conceptual Framework

**Figure 1:**
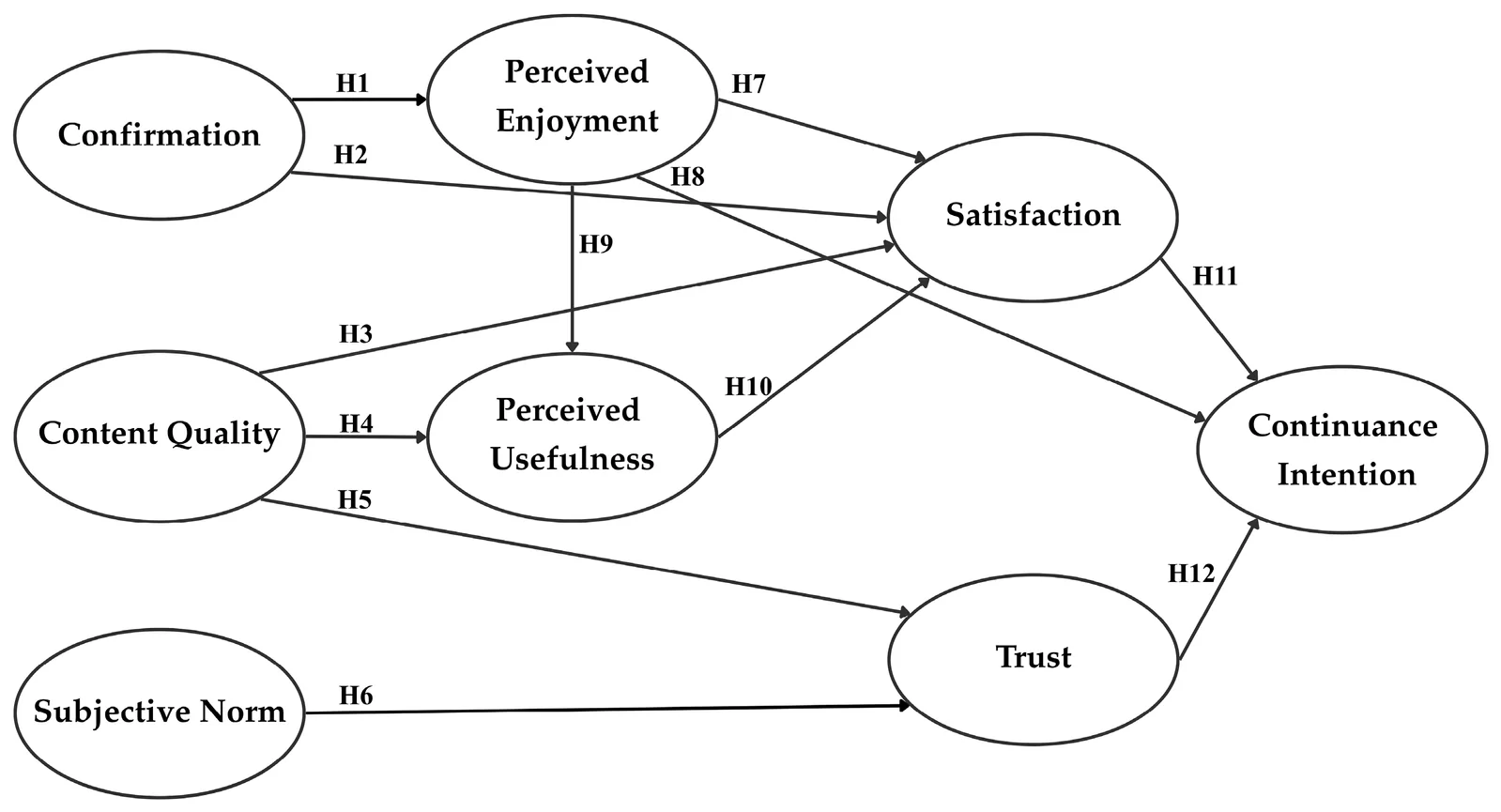
The proposed research model.

Figure 1 presents the proposed model, in which confirmation, content quality, and subjective norm are positioned as antecedents; perceived enjoyment, perceived usefulness, satisfaction, and trust operate as post-adoption evaluations or mechanisms; and continuance intention is the outcome. Drawing from the logic of the ECM, CON is positioned as a key post-adoption determinant influencing both PE (H1) and SAT (H2). While the original ECM does not explicitly incorporate affective constructs, prior studies within extended ECM frameworks suggest that CON may also enhance PE, reflecting the affective dimension of post-adoption evaluation (Bhattacherjee, 2001; Huang & Liu, 2024). In addition, CQ, derived from the DeLone and McLean Information Systems Success Model, is hypothesized to influence SAT (H3), PU (H4), and TRU (H5) (DeLone & McLean, 2003; Cho et al., 2023). Furthermore, SN, based on the Theory of Planned Behavior, is proposed to affect TRU (H6), as prior research in digital contexts suggests that social influence from significant others may contribute to the development of users’ trust in online platforms (Ajzen, 1991; Rahmayanti et al., 2022). Within the internal mechanism, PE plays a significant role in shaping both affective and behavioral responses. It is hypothesized to influence SAT (H7), CI (H8), and PU (H9), reflecting the affective role of enjoyment in online and technology-mediated learning contexts (Davis et al., 1992; Huang & Liu, 2024). PU further contributes to SAT (H10), reinforcing the cognitive evaluation process. Finally, SAT (H11) and TRU (H12) are proposed as key determinants of CI. Prior studies suggest that TRU has a significant influence on CI, either directly or indirectly through mediating mechanisms, depending on the context of digital platform usage (Gefen et al., 2003; D. M. Nguyen et al., 2021; Rahmayanti et al., 2022).

## 3. Research Methods

A sequential exploratory mixed-methods design was used in this study (Creswell & Plano Clark, 2018), beginning with a qualitative phase for instrument development followed by a quantitative phase for model testing.

### 3.1. Qualitative Research: Instrument Development with Fuzzy E-Delphi

**Figure 2:**
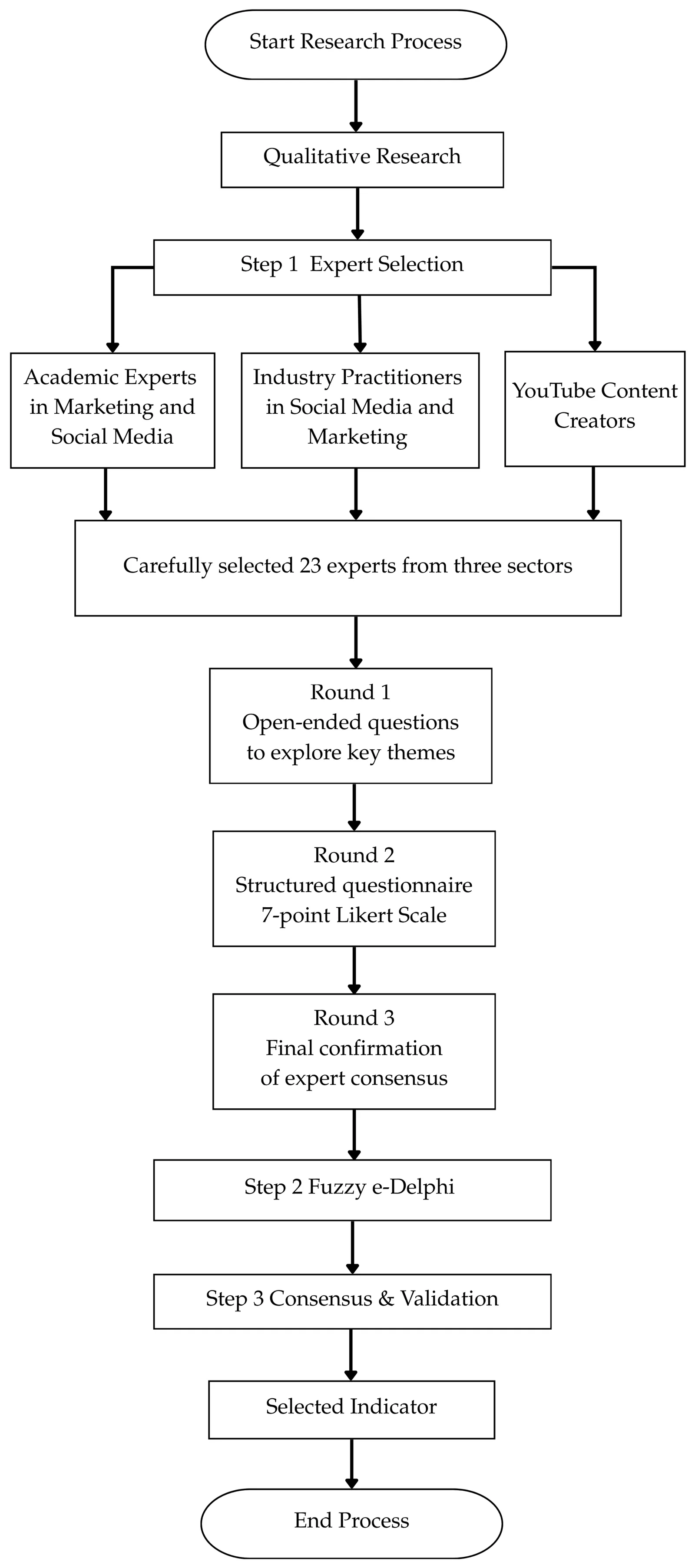
A qualitative research framework based on the Fuzzy e-Delphi Method.

The initial phase aimed to develop and validate a robust measurement instrument. We used the Fuzzy e-Delphi Method, a technique that systematically aggregates expert opinions while managing the inherent ambiguity in linguistic judgments (Ishikawa et al., 1993). This method is particularly useful for building consensus in educational technology research (Hasim et al., 2023). The overall qualitative research process is illustrated in Figure 2.

#### 3.1.1. Population and Sampling

For this phase, the target group comprised individuals with expertise in digital learning, social media, marketing, and content creation. Using purposive sampling, a panel of 23 experts was selected from three professional groups to ensure a balanced range of perspectives. Eligibility required at least three years of relevant experience. The first group consisted of seven full-time university lecturers with knowledge and expertise in marketing and social media and at least three years of professional experience. The second group comprised eight practitioners working in organizations related to social media platforms or marketing, each with at least three years of professional experience. The third group consisted of eight YouTube channel owners whose channels had been active for at least three years and had more than 100,000 subscribers. The panel size exceeded the minimum of 17 experts suggested by Macmillan (1971), supporting a high level of reliability in the consensus process.

#### 3.1.2. Research Instruments

Data were collected over three iterative rounds using an online questionnaire. In Round 1, an open-ended survey was used to gather broad insights from the experts. For Round 2, a structured questionnaire was developed using themes from the first round of analysis and evidence from prior studies, where experts rated indicators on a 7-point Likert scale. Round 3 involved a final closed-ended questionnaire to confirm the consensus.

#### 3.1.3. Data Collection

The e-Delphi process included three rounds and was carried out from October to December 2025. The questionnaire forms were sent to the expert panel by email, allowing all participants to respond through the same channel and within the same procedure. In Round 1, open-ended questions invited experts to comment on construct coverage, the clarity of indicators, and points that needed improvement. Their responses were examined through content analysis and then used to prepare the questionnaire for the next round. In Round 2, experts rated the revised items using a seven-point Likert scale, ranging from 1 = strongly irrelevant to 7 = strongly relevant, to judge item relevance. In Round 3, summarized feedback and group-level results were returned to the experts, allowing them to reconsider their ratings and confirm the level of consensus across the items.

#### 3.1.4. Data Analysis

**Figure 3:**
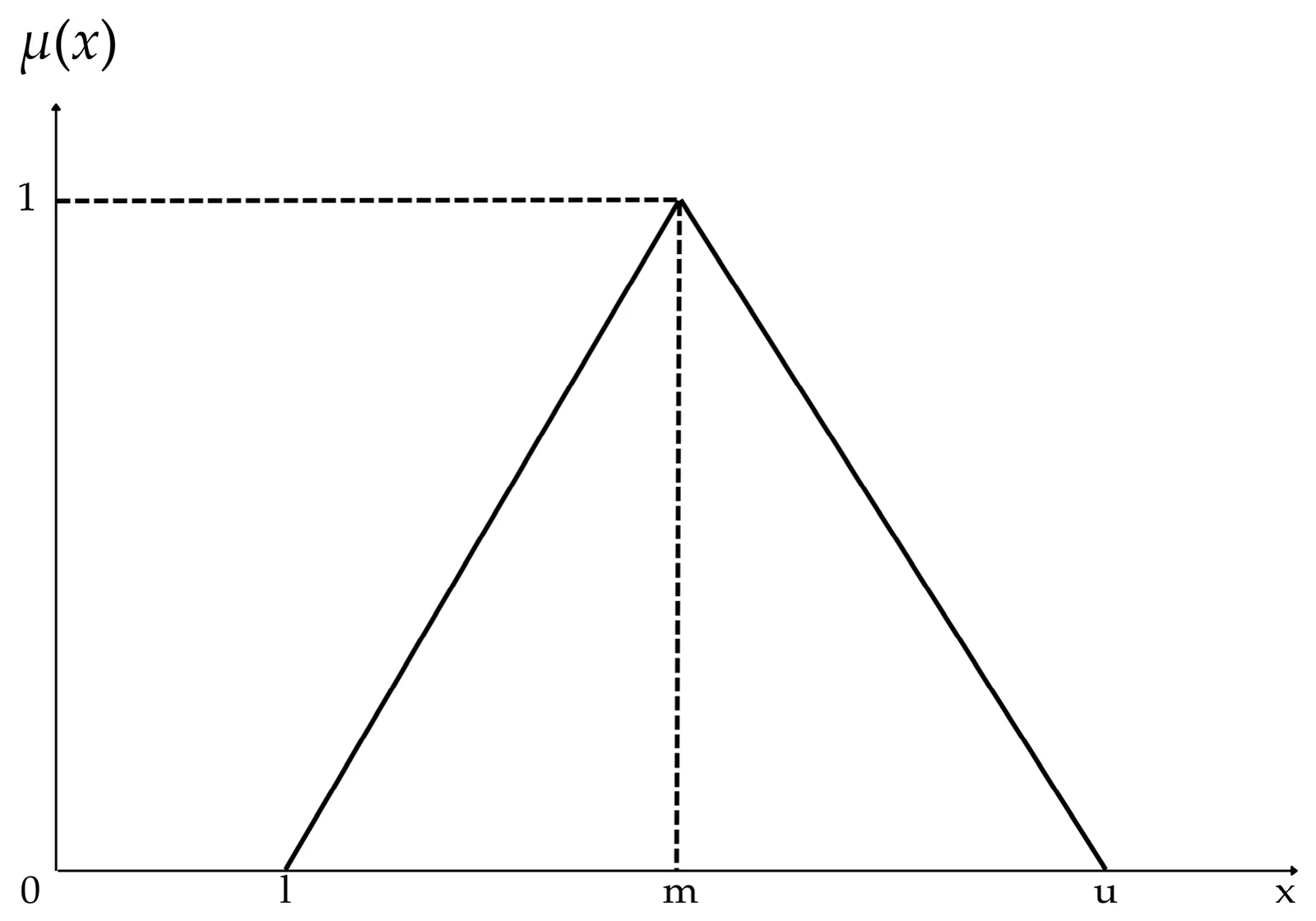
Triangular Fuzzy Number (TFN).

The Fuzzy e-Delphi Method (FDM) takes the traditional Delphi technique a step further by bringing in fuzzy set theory to handle the messy reality of expert opinions (Ishikawa et al., 1993). Unlike the conventional approach that forces experts into rigid, crisp values, FDM uses a triangular TFN. As illustrated in Figure 3, a TFN is defined by three points (l, m, u), indicating the minimum value, the most promising value, and the highest possible value, respectively (Zadeh, 1965). This shift allows researchers to translate vague, linguistic feedback into precise mathematical terms, which actually explains the true level of consensus much better (Benítez et al., 2007). By mixing human expertise with strict mathematical aggregation, the method cuts through personal bias and makes the final decision far more objective (Habibi et al., 2015).

In this study, a 7-point Likert scale was used to collect expert ratings. Each scale value was then converted into its corresponding TFN parameters, as shown in Table 1.

First, during the fuzzification stage, we converted the experts’ linguistic ratings into TFNs based on the conversion scheme in Table 1. Next, we aggregated these individual fuzzy scores to calculate a single fuzzy mean for each item. The third step involved defuzzification, where the aggregated fuzzy values were transformed into crisp scores using the center of gravity method:

Crisp Score = (l + m + u)/3

Indicators with defuzzified scores equal to or greater than 0.83 were retained, whereas those scoring below 0.83 were to be discarded. This threshold was employed to ensure a high level of expert agreement and was more stringent than the conventional 0.70–0.80 range commonly adopted in FDM research (Habibi et al., 2015). Recent empirical applications further support the adoption of this elevated criterion (Kiatsangsilp & Pankham, 2025).

### 3.2. Quantitative Research

**Figure 4:**
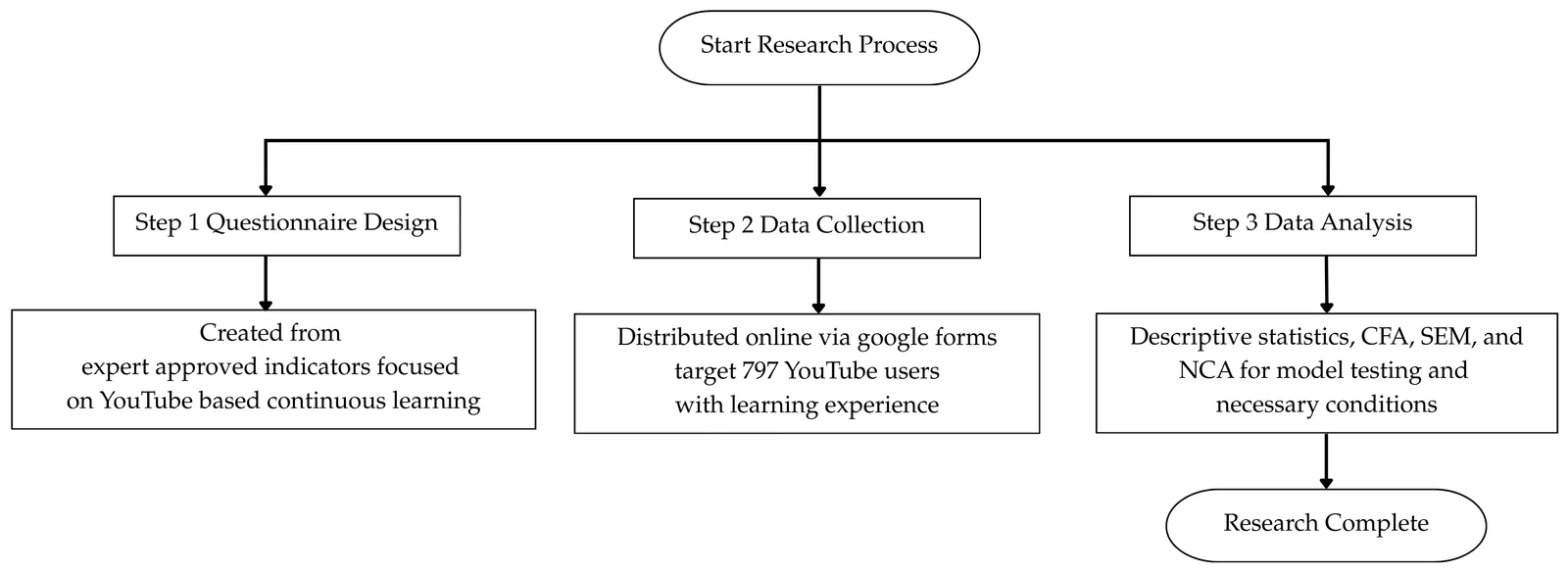
Quantitative research framework.

In Phase 2, the model validated by experts was empirically examined using a large sample of YouTube learners in Thailand. As shown in Figure 4, the quantitative research process followed three sequential steps.

#### 3.2.1. Population and Sampling

The target population comprised Thai residents aged 18 years or older who had used YouTube for informal learning during the preceding six months. Respondents were recruited through nonprobability purposive online sampling via educational YouTube channels and related Facebook community pages. Eligibility was verified through screening questions before respondents were permitted to access the main questionnaire. A total of 797 eligible and complete responses were included in the analysis. The demographic and background characteristics of the respondents are presented in Section 4.

#### 3.2.2. Research Instruments

The survey instrument included demographic questions and measurement items for the eight latent constructs, modified from previously validated scales. Responses were recorded using a seven-point Likert scale (1 = Strongly disagree to 7 = Strongly agree). The measurement items were developed based on established scales from the literature and adapted to the context of YouTube-based informal learning. The operational definitions for each construct are presented in Table 2.

Internal consistency was examined through Cronbach’s alpha (α) and Composite Reliability (CR). For both indices, scores at or above 0.70 were treated as evidence of acceptable reliability. Convergent validity was then checked through the Average Variance Extracted (AVE), where a threshold of 0.50 or above was used as the criterion (Hair et al., 2019).

#### 3.2.3. Data Collection

Before accessing the main questionnaire, prospective respondents completed three eligibility screening questions. Respondents were required to confirm that they were at least 18 years old, had used YouTube for learning during the preceding six months, and resided in Thailand. Residence was identified by selecting one of six regions of Thailand, namely Central, Northern, Eastern, Northeastern, Western, or Southern Thailand. Only respondents who met all three criteria were permitted to proceed to the main questionnaire. Those who did not meet any criterion were automatically directed to the end of the questionnaire. Ethical approval was obtained from the Ethics Review Board of Rangsit University (COA No. RSUERB2025-141; 23 June 2025). Before participation, respondents reviewed information about the study and provided electronic informed consent. Participation was voluntary and anonymous, and respondents could discontinue the questionnaire at any time before submission.

#### 3.2.4. Analysis of Data

The dataset was first checked for incomplete responses, extreme values, and distributional normality. No incomplete responses or item-level missing data were identified among the eligible submissions; therefore, the final analytical dataset comprised 797 complete responses. Normality was assessed by confirming that skewness and kurtosis values fell within ±3.0 (Kline, 2011). Collinearity was assessed using VIFs, with values below 5.00 considered acceptable (Hair et al., 2019). Data screening and preliminary and descriptive analyses were conducted using IBM SPSS Statistics version 29.0. Confirmatory factor analysis and structural equation modeling were performed using IBM SPSS Amos version 26.0, whereas Necessary Condition Analysis was conducted using SmartPLS version 4.1.1.8. The measurement model was assessed through CFA, after which the structural model was tested to evaluate the proposed relationships among the study variables. Discriminant validity was assessed using HTMT (<0.85; Hair et al., 2019). The model fit was evaluated against several widely accepted criteria: CMIN/df ≤ 3.00, AGFI ≥ 0.90, and GFI ≥ 0.90 (Hair et al., 2019); NFI ≥ 0.90 (Bentler & Bonett, 1980); and CFI ≥ 0.90, TLI ≥ 0.90, RMSEA < 0.08, and SRMR < 0.08 (Hu & Bentler, 1999). Specific indirect effects were tested using 5000 bootstrap resamples and 95% confidence intervals; effects were significant when the intervals excluded zero. After SEM, NCA was further employed to identify whether selected determinants functioned as necessary conditions for achieving high continuance intention. The NCA results were interpreted using effect size, permutation-based significance testing, and bottleneck analysis, following recent methodological recommendations (Dul et al., 2020, 2023).

## 4. Results

### 4.1. Qualitative Results

All 33 items met the prespecified Fuzzy e-Delphi retention criterion of 0.83, with crisp scores ranging from 0.854 to 0.956; therefore, all items were retained for the quantitative survey (Ishikawa et al., 1993; Macmillan, 1971). The results are detailed in Table 3.

### 4.2. Quantitative Results

#### 4.2.1. Descriptive Statistics

The final sample comprised 797 respondents. The largest groups were women (61.0%), respondents aged 18–24 years (39.6%), bachelor’s-degree holders (41.7%), government or state-enterprise employees (38.8%), students (37.4%), and residents of Central Thailand (65.7%) The results are detailed in Table 4.

#### 4.2.2. Measurement Model Analysis: Reliability and Convergent Validity

The measurement model demonstrated acceptable reliability and convergent validity shown in Table 5. Standardized loadings ranged from 0.710 to 0.960, Cronbach’s alpha ranged from 0.840 to 0.946, composite reliability ranged from 0.853 to 0.943, and average variance extracted ranged from 0.594 to 0.806; all values met the recommended criteria (Hair et al., 2019).

#### 4.2.3. Common Method Bias Assessment

Given the single-source, cross-sectional, self-reported nature of the data, common method bias was assessed using full-collinearity VIFs (Kock, 2015). As shown in Table 5. the VIF values ranged from 1.701 to 4.266 and were all below the general collinearity threshold of 5.00 (Hair et al., 2021). Although perceived enjoyment (VIF = 3.393) and satisfaction (VIF = 4.266) exceeded the conservative threshold of 3.30, the results did not indicate severe collinearity or pervasive common method bias across the model.

#### 4.2.4. Goodness of Fit of the Measurement Model

To further assess the measurement model’s robustness, goodness-of-fit indices were examined for both the individual construct CFAs and the full measurement model. The results are summarized in Table 6, with the full measurement model used as the primary basis for evaluating overall model fit. The full measurement model yielded a CMIN/df value of 2.620, which was below the recommended maximum of 3.0 (Hair et al., 2019). Other key indices, including CFI (0.969), NFI (0.951), and TLI (0.964), exceeded the recommended threshold of 0.90 (Hu & Bentler, 1999; Bentler & Bonett, 1980). Moreover, the RMSEA value of 0.045 and SRMR value of 0.029 were below the recommended limits of 0.06 and 0.05, respectively (Hu & Bentler, 1999; Hair et al., 2019). Taken together, these results support the acceptable fit of the full measurement model before evaluating the structural relationships.

#### 4.2.5. Discriminant Validity

As shown in Table 7, all HTMT values ranged from 0.415 to 0.829 and remained below 0.85, supporting discriminant validity among the eight constructs (Henseler et al., 2015).

### 4.3. Structural Model Assessment and Hypothesis Results

#### 4.3.1. Structural Model Fit and Hypothesis Testing

The structural model showed acceptable fit (CMIN/df = 2.546, AGFI = 0.902, GFI = 0.922, CFI = 0.971, NFI = 0.954, TLI = 0.966, RMSEA = 0.044, and SRMR = 0.033), as shown in Table 8 (Hair et al., 2019; Hu & Bentler, 1999).

**Figure 5:**
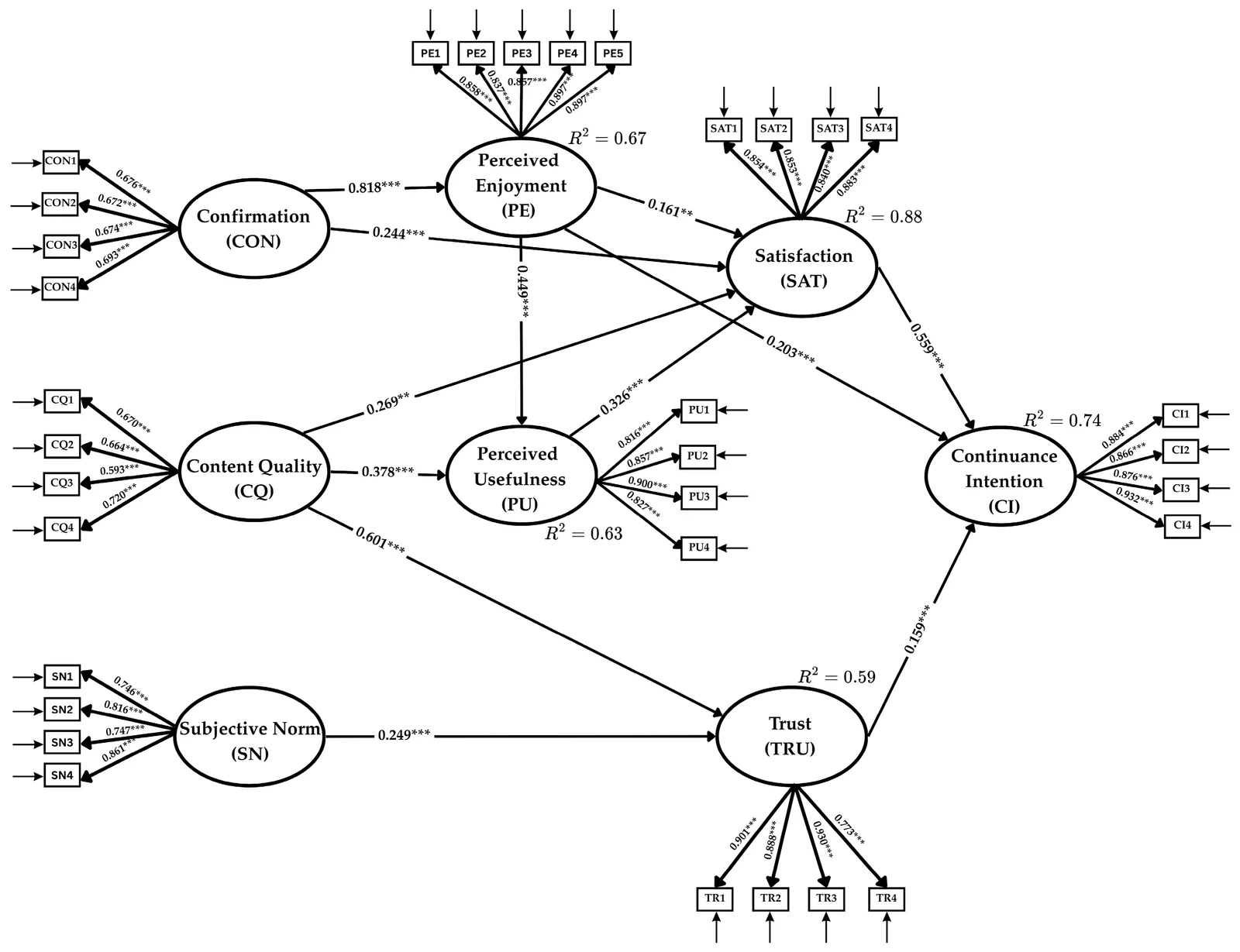
Final SEM model with standardized path estimates. *p* < 0.01 = **; *p* < 0.001 = ***.

The model explained 67% of the variance in perceived enjoyment, 63% of the variance in perceived usefulness, 88% of the variance in satisfaction, 59% of the variance in trust, and 74% of the variance in continuance intention show in Figure 5. All 12 hypothesized paths were significant; satisfaction was the strongest direct predictor of continuance intention (β = 0.559, *p* < 0.001), followed by perceived enjoyment (β = 0.203, *p* < 0.001) and trust (β = 0.159, *p* < 0.001) (Table 9).

#### 4.3.2. Mediation Analysis: Bootstrapped Specific Indirect Effects

To examine the mechanisms through which the antecedent constructs influenced continuance intention, the specific indirect effects were assessed using a nonparametric bootstrapping procedure with 5000 resamples and 95% confidence intervals. An indirect effect was considered statistically significant when its confidence interval did not include zero. The results are presented in Table 10.

As shown in Table 10, all eight specific indirect effects were statistically significant because none of the 95% bootstrap confidence intervals included zero. Confirmation influenced continuance intention through four significant mediating pathways. The strongest of these operated through perceived enjoyment (β = 0.166, 95% CI: [0.060, 0.272]), followed by satisfaction (β = 0.136, 95% CI: [0.039, 0.234]). The serial pathways through PE and SAT (β = 0.074, 95% CI: [0.017, 0.131]) and through PE, PU, and SAT (β = 0.067, 95% CI: [0.030, 0.104]) were also significant. CQ influenced CI: through SAT (β = 0.150, 95% CI: [0.022, 0.279]), TRU (β = 0.096, 95% CI: [0.053, 0.138]), and the sequential pathway through PU and SAT (β = 0.069, 95% CI: [0.026, 0.112]). Finally, SN had a significant indirect effect on CI: through TRU (β = 0.040, 95% CI: [0.018, 0.062]). This result supports the mediating role of trust in transmitting the influence of SN to learners’ CI.

### 4.4. NCA Results

**Figure 6:**
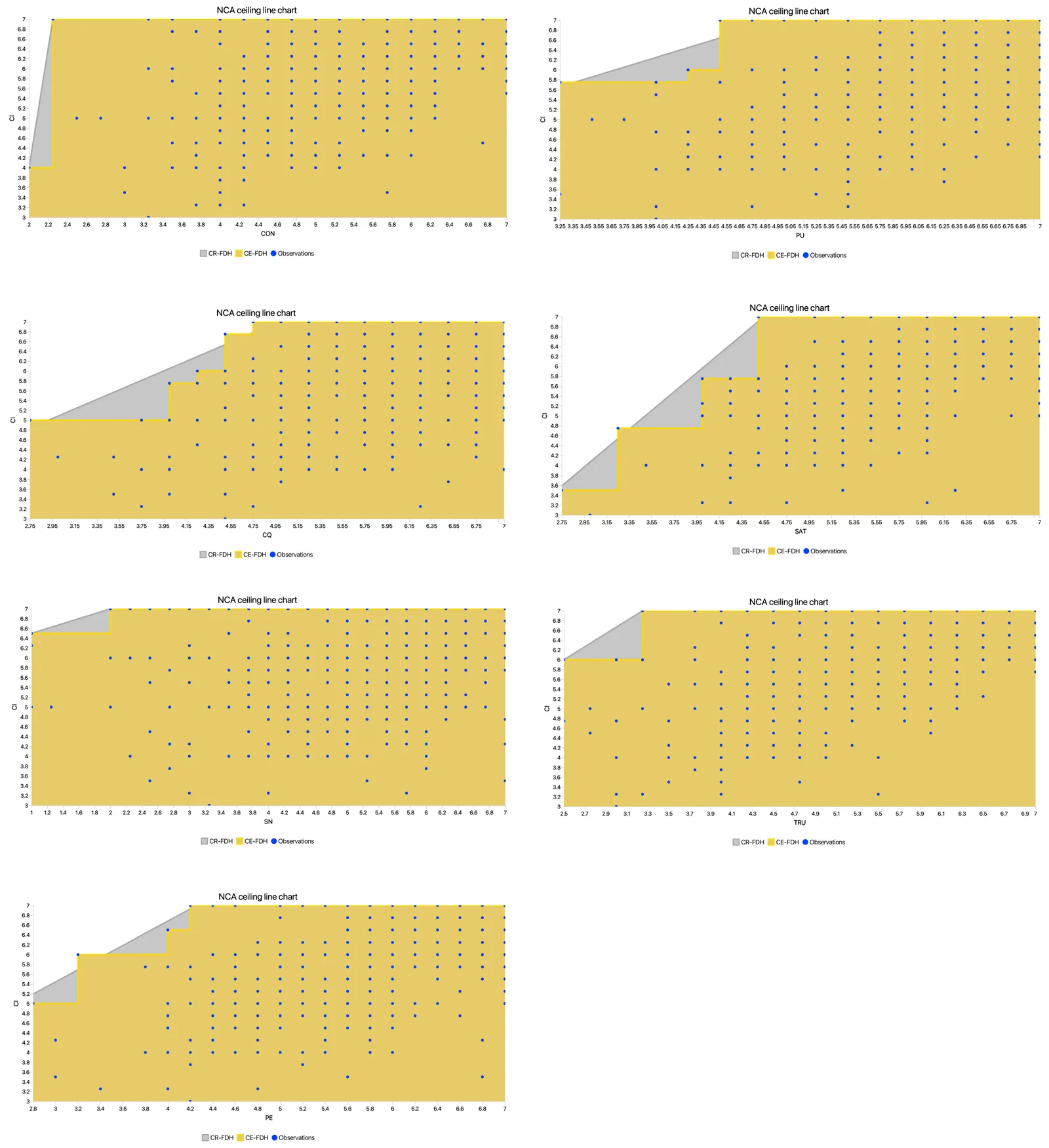
NCA ceiling line charts.

NCA was used to evaluate confirmation, content quality, subjective norm, perceived enjoyment, perceived usefulness, satisfaction, and trust as potential lower-bound conditions for high continuance intention (Figure 6). The necessity relationships identified by NCA represent empirical patterns within the observed sample and should not be interpreted as evidence of causal necessity.

The panels display the CE-FDH and CR-FDH ceiling lines based on mean composite scores for each antecedent of continuance intention. Larger empty spaces near the upper-left ceiling area indicate stronger necessary-condition patterns. Detailed effect sizes and bottleneck values are shown in Table 11 and Table 12.

Satisfaction showed the largest necessity effect under CE-FDH (d = 0.239, *p* < 0.001) and CR-FDH (d = 0.180, *p* < 0.001), followed by content quality (d = 0.184 and 0.142, respectively; both *p* < 0.001). Perceived enjoyment and perceived usefulness showed smaller significant effects, whereas confirmation and subjective norm were not necessary conditions. Trust showed only a very small CE-FDH effect, and its CR-FDH effect was nonsignificant.

At the maximum continuance-intention target (7.000), the CE-FDH bottleneck values were 4.750 for content quality, 4.500 for perceived usefulness, 4.500 for satisfaction, and 4.200 for perceived enjoyment. The corresponding CR-FDH values were 2.250 for confirmation, 4.979 for content quality, 2.000 for subjective norm, 4.255 for perceived enjoyment, 4.955 for perceived usefulness, 4.548 for satisfaction, and 3.250 for trust. Both techniques indicated that satisfaction and content quality formed the strongest lower-bound constraints, with perceived usefulness and perceived enjoyment as smaller supporting conditions. The apparent increase in the subjective norm bottleneck value at the 90% and 100% CI targets reflects the stepwise nature of the CE-FDH ceiling line and the observed score distribution. However, because the overall necessity effect of subjective norm was small and statistically nonsignificant, this isolated bottleneck value is not interpreted as evidence that subjective norm is a necessary condition.

## 5. Discussion

This study developed and tested a layered model explaining Thai learners’ continuance intention toward YouTube for informal learning. The constructs and indicators were refined through the Fuzzy e-Delphi process before the proposed relationships were examined using SEM and NCA. SEM identified the predictive relationships among the constructs, whereas NCA identified empirical minimum conditions associated with high levels of CI (Dul, 2016; Dul et al., 2023). The combined findings distinguish predictors that contribute to CI from empirical conditions that may constrain its achievement at high levels. The following sections discuss these findings in relation to prior research, theory, practice, and future research.

### 5.1. Discussion of Key Findings

Regarding RQ1, the SEM results indicate that satisfaction, perceived enjoyment, and trust are the direct predictors of continuance intention, whereas confirmation, content quality, subjective norm, and perceived usefulness contribute through the hypothesized structural relationships. The bootstrapped indirect-effect analysis further clarifies these mechanisms. Regarding RQ2, the NCA results identify satisfaction and content quality as the principal necessary conditions, with perceived enjoyment and perceived usefulness playing smaller supporting roles.

Satisfaction emerged as the central determinant of CI. It was the strongest direct predictor in SEM and the strongest necessary condition in NCA, reinforcing the central role of satisfaction in the ECM and in previous continuance research (Bhattacherjee, 2001; D. M. Nguyen et al., 2021). This finding is also consistent with evidence from YouTube learning contexts showing that favorable post-use evaluations support continued use (Sukma Ningsih et al., 2024). Taken together, the results indicate that satisfaction strengthens CI while also establishing an important empirical minimum condition for its highest levels. CON was positively associated with PE and SAT. Its strongest relationship was with perceived enjoyment, which is consistent with prior studies showing that fulfilled expectations support positive affective evaluations in online learning environments (Al-Maroof et al., 2021; Inan et al., 2023). PE, in turn, contributed to perceived usefulness, satisfaction, and CI. This pattern indicates that affective and cognitive evaluations are interconnected in informal video learning (Huang & Liu, 2024). Content quality was positively associated with satisfaction, perceived usefulness, and trust, and its indirect relationships with CI operated through these constructs. The NCA results further identified content quality as one of the strongest necessary conditions. These findings are consistent with previous research emphasizing the importance of high-quality information and learning content on open video platforms (Cho et al., 2023; Shang & Lyv, 2022). SN was positively associated with trust, and the specific indirect effect from SN to CI through trust was statistically significant. These findings are consistent with previous studies linking social influence with trust and subsequent behavioral outcomes in digital contexts (Barattucci et al., 2022; Cicin & Çetin Gürkan, 2026). However, subjective norm was not supported as a necessary condition in the NCA. TRU significantly predicted CI in SEM and mediated the indirect relationships of CQ and SN with CI. Nevertheless, its necessity effect was small and was not consistently supported across both ceiling line techniques. TRU is therefore better interpreted as a supportive relational predictor within the structural pathways than as a foundational necessary condition for high CI. PE had a positive relationship with PU, supporting the view that affective experience can shape cognitive evaluation in voluntary learning settings (Huang & Liu, 2024). Although PE and PU had smaller necessary effects than SAT and CQ, both functioned as supporting minimum conditions for high CI. These necessity findings should be interpreted by considering effect size, statistical significance, and bottleneck levels together (Dul et al., 2020, 2023). The positive structural relationships observed in this voluntary setting should also be understood as context dependent, given that different patterns have been reported in formal learning settings (J. Li et al., 2025). Beyond the direct and necessity-based findings, the bootstrapped specific indirect effects further clarify the mechanisms associated with learners’ CI. All eight specific indirect effects were statistically significant, as none of their 95% bootstrap confidence intervals included zero. CON was indirectly associated with CI through PE and SAT, as well as through the serial pathways involving PE and SAT and involving PE, PU, and SAT. These findings indicate that the fulfillment of prior expectations is associated with continued learning through interconnected affective and cognitive evaluations. CQ was also indirectly associated with CI through SAT, TRU, and the sequential pathway involving PU and SAT. This pattern suggests that CQ supports continued engagement through both favorable post-use evaluation and credibility formation. Finally, the significant SN → TRU → CI pathway provides formal statistical support for the social-validation mechanism discussed above: SN is associated with continuance intention through its relationship with TRU. However, this pathway should be interpreted only as an indirect association among the measured constructs and not as evidence of a broader cultural or causal mechanism.

### 5.2. Contributions to Theory

The findings reinforce the ECM as a useful foundation for explaining CI in informal online learning (Bhattacherjee, 2001). Integrating CQ (DeLone & McLean, 2003), SN (Ajzen, 1991), and TRU (Gefen et al., 2003) extends the model by incorporating informational and relational determinants relevant to open social media platforms. The significant indirect relationship from SN to CI through TRU indicates that social influence can operate through a relational pathway rather than only through a direct relationship with behavioral intention. This interpretation is consistent with recent evidence concerning the relationship between SN and TRU in digital contexts (Cicin & Çetin Gürkan, 2026). A further contribution comes from combining SEM with NCA. The SEM findings identify net predictive relationships within the proposed model, whereas the NCA findings identify empirical minimum conditions for high CI. SAT and CQ emerged as the principal necessary conditions, while PE and PU played supporting roles. CON and SN did not exhibit statistically supported necessity effects despite their significant roles within the structural pathways. Trust similarly contributed to CI in SEM but showed only a weak and less robust necessity effect. These differences demonstrate that a significant predictor is not necessarily a foundational necessary condition and provide a more differentiated explanation of CI. The findings also extend technology acceptance research by showing that PE contributes to PU in voluntary informal learning. This result indicates that positive affective evaluations can shape learners’ cognitive assessments of platform value. The pattern observed in this informal setting also differs from findings reported in some formal learning contexts, indicating that the roles of cognitive and relational evaluations may depend on the learning environment (J. Li et al., 2025).

### 5.3. Practical Implications

For content creators and educational influencers, the findings emphasize the importance of producing relevant, current, diverse, and inspiring learning content. CQ contributes to SAT, PU, and TRU, while SAT is the most prominent determinant of CI. Creators should therefore combine reliable content with an enjoyable learning experience that meets or exceeds learners’ expectations. Recommendations from peers, experts, and other important people may support trust, but they should complement rather than replace demonstrable CQ.

For platform developers, the results emphasize convenient access, flexible learning, replay functionality, transparent data practices, and features that support a satisfactory user experience. TRU remains relevant because it contributes to CI, although the NCA findings indicate that its role is supportive rather than foundational. Platform improvements should therefore balance credibility and privacy measures with the stronger priorities of CQ, PU, PE, and SAT.

For educational institutions, the findings support the use of YouTube as a supplementary resource for informal and lifelong learning. Institutions should strengthen learners’ ability to evaluate the relevance, currency, diversity, and credibility of online content. The NCA results may also be used diagnostically within the present context. SAT and CQ should receive the highest priority, followed by PU and PE. The reported bottleneck values are sample dependent and should guide context specific monitoring rather than be treated as universal cutoff scores.

### 5.4. Limitations and Suggestions for Future Research

Several limitations should be considered. First, the cross-sectional design captures perceptions at one point in time and does not establish causality. The NCA findings likewise represent empirical lower bound patterns within the observed dataset rather than causal necessity. Future research could use longitudinal designs to examine changes in perceptions and CI over time. Second, many respondents were recruited from educational channels and online communities. This strategy provided access to respondents with relevant YouTube learning experience but may have increased baseline levels of SAT and TRU. Future studies should use broader sampling strategies across different channels and user groups. Third, the structural model did not include demographic or usage-related control variables, such as age, gender, and frequency of YouTube use, because the analysis was designed to examine the theoretically specified relationships among the focal constructs. Consequently, potential heterogeneity associated with these respondent characteristics cannot be ruled out. Future research should incorporate relevant control variables and, where theoretically appropriate, conduct multigroup or moderation analyses to assess the robustness of the proposed relationships across different demographic and usage-based subgroups. Finally, the study was conducted with YouTube users in Thailand. Replication with samples from other countries, platforms, and learning settings would help assess the generalizability of the proposed relationships and necessary conditions.

## 6. Conclusions

This study examined Thai learners’ continuance intention to use YouTube for informal learning by extending the expectation–confirmation model to include content quality, subjective norm, perceived enjoyment, and trust. The structural results showed that satisfaction was the strongest direct predictor of continuance intention. Confirmation, content quality, perceived enjoyment, perceived usefulness, and trust contributed through interconnected structural relationships. The NCA results complemented these findings by identifying satisfaction and content quality as the principal necessary conditions for achieving high continuance intention, with perceived usefulness and perceived enjoyment functioning as smaller supporting conditions. The structural results also showed that subjective norm was associated with trust, which was in turn associated with continuance intention. The bootstrapped mediation analysis further supported this mechanism, demonstrating a statistically significant indirect effect of SN on CI through TRU. Practically, the findings suggest that educators, content creators, and platform designers should prioritize high-quality content and satisfactory learning experiences while supporting perceived usefulness, enjoyment, and trust. Overall, the combined SEM–NCA evidence clarifies both the relational mechanisms and the minimum conditions underlying learners’ continuance intention, providing an evidence-based foundation for developing credible, satisfying, and sustainable informal learning environments on YouTube.

## Figures and Tables

**Table 1:** TFN scale for 7-point Likert response measurement.

Scale Value	Linguistic Variable	TFN Parameters (l, m, u)
1	Least Appropriate	(0.00, 0.00, 0.17)
2	Slightly Appropriate	(0.00, 0.17, 0.33)
3	Moderately Inappropriate	(0.17, 0.33, 0.50)
4	Neutral	(0.33, 0.50, 0.67)
5	Moderately Appropriate	(0.50, 0.67, 0.83)
6	Very Appropriate	(0.67, 0.83, 1.00)
7	Most Appropriate	(0.83, 1.00, 1.00)

**Table 2:** Operational definitions of research constructs.

Construct	Abbreviation	Operational Definition	Adapted From
Confirmation	CON	The degree to which a user’s initial expectations of YouTube for learning are met or exceeded after actual use.	Bhattacherjee (2001)
Content Quality	CQ	The perceived relevance, timeliness, diversity, and inspirational value of educational content available on YouTube.	DeLone and McLean (2003)
Subjective Norm	SN	The influence learners feel from important people around them (e.g., peers, instructors, family) to use YouTube for learning.	Ajzen (1991)
Perceived Usefulness	PU	The extent to which a learner views YouTube as usefully providing convenience, flexibility, and efficiency in their learning.	Davis (1989); Bhattacherjee (2001)
Perceived Enjoyment	PE	The degree to which learners find YouTube-based learning enjoyable, engaging, and personally rewarding.	Davis et al. (1992)
Satisfaction	SAT	The user’s overall affective evaluation and feeling of contentment resulting from their YouTube learning experience.	Bhattacherjee (2001)
Trust	TRU	The user’s belief in the reliability, integrity, and credibility of the YouTube platform and its educational content.	Gefen et al. (2003)
Continuance Intention	CI	The user’s subjective willingness to continue using YouTube as a learning platform in the future.	Bhattacherjee (2001)

**Table 3:** Expert panel consensus derived from the FDM.

Construct	Item Code	Question	Crisp Value ≥ 0.83	Result
Confirmation (CON)	CON1	Using YouTube as a learning platform exceeded my expectations	0.854	Validated
	CON2	The system of the YouTube platform for learning exceeded my expectations	0.876	Validated
	CON3	The learning content on YouTube exceeded my expectations	0.896	Validated
	CON4	My experience of using YouTube for learning exceeded my expectations	0.887	Validated
Content Quality (CQ)	CQ1	The learning content on YouTube is relevant	0.923	Validated
	CQ2	The learning content on YouTube is up to date	0.920	Validated
	CQ3	The learning content on YouTube is diverse	0.923	Validated
	CQ4	The learning content on YouTube is inspiring	0.927	Validated
Subjective Norm (SN)	SN1	My friends influence my use of YouTube for learning	0.893	Validated
	SN2	Experts influence my use of YouTube for learning	0.896	Validated
	SN3	Celebrities influence my use of YouTube for learning	0.930	Validated
	SN4	Most people who are important to me affect my decision to use YouTube for learning.	0.867	Validated
Perceived Enjoyment (PE)	PE1	I like using YouTube for learning	0.941	Validated
	PE2	I enjoy using YouTube for learning	0.938	Validated
	PE3	I have fun using YouTube for learning	0.934	Validated
	PE4	I feel comfortable using YouTube for learning	0.930	Validated
	PE5	I am happy when I use YouTube for learning	0.901	Validated
Perceived Usefulness (PU)	PU1	Using YouTube for learning allows me to study anywhere	0.927	Validated
	PU2	Using YouTube for learning is convenient	0.923	Validated
	PU3	Using YouTube for learning allows me to learn quickly	0.883	Validated
	PU4	Using YouTube for learning allows me to replay content at any time	0.912	Validated
Satisfaction (SAT)	SAT1	I am satisfied with the system of the YouTube platform for learning	0.949	Validated
	SAT2	I am satisfied with the learning content available on YouTube	0.956	Validated
	SAT3	I am satisfied with my engagement in using the YouTube platform for learning	0.949	Validated
	SAT4	I am satisfied with my experience of using the YouTube platform for learning	0.952	Validated
Trust (TRU)	TRU1	I trust the reputation of the content creators on YouTube for learning	0.923	Validated
	TRU2	I trust the recommendations provided by content creators on YouTube for learning	0.923	Validated
	TRU3	I trust the quality of content on YouTube for learning	0.909	Validated
	TRU4	I trust the privacy and security of my personal data on YouTube for learning	0.930	Validated
Continuance Intention (CI)	CI1	I am likely to continue using YouTube for learning	0.941	Validated
	CI2	I will continue to use YouTube for learning	0.938	Validated
	CI3	I intend to continue using YouTube for learning	0.934	Validated
	CI4	I am willing to continue using YouTube for learning	0.930	Validated

**Table 4:** Demographic and background information of respondents.

Characteristic	Category	Frequency	Percentage (%)
Gender	Male	295	37.0%
	Female	486	61.0%
	Other	16	2.0%
Age (years)	18–24	316	39.6%
	25–34	137	17.2%
	35–44	164	20.6%
	45–54	113	14.2%
	55 Above	67	8.4%
Highest level of education	Less than bachelor’s degree	276	34.6%
	Bachelor’s qualification	332	41.7%
	Master’s qualification	164	20.6%
	Doctorate	25	3.1%
Occupation	Government officer/state enterprise staff	309	38.8%
	Private company employee	55	6.9%
	Student	298	37.4%
	Business owner	43	5.4%
	Freelancer	62	7.8%
	Other	30	3.7%
Region	Central	524	65.7%
	Northern	90	11.3%
	Eastern	22	2.8%
	Northeastern	112	14.1%
	Western	6	0.8%
	Southern	43	5.3%
Learning category	Professional skills and business	84	10.5%
	Academic knowledge and language	136	17.1%
	Technology and digital skills	167	21.0%
	Hobbies and personal interests	246	30.9%
	Social skills and self-development	74	9.3%
	Health and well-being	64	8.0%
	Other	26	3.2%

**Table 5:** Measurement model results.

Variable	Measurement Item	Factor Loading	Cronbach’s α	CR	AVE	VIF
Confirmation (CON)			0.927	0.927	0.763	2.156
	CON1	0.750				
	CON2	0.810				
	CON3	0.960				
	CON4	0.960				
Content Quality (CQ)			0.840	0.853	0.594	2.552
	CQ1	0.731				
	CQ2	0.890				
	CQ3	0.710				
	CQ4	0.740				
Subjective Norm (SN)			0.862	0.872	0.631	1.701
	SN1	0.820				
	SN2	0.750				
	SN3	0.820				
	SN4	0.790				
Perceived Enjoyment (PE)			0.937	0.939	0.756	3.393
	PE1	0.830				
	PE2	0.880				
	PE3	0.900				
	PE4	0.870				
	PE5	0.870				
Perceived Usefulness (PU)			0.910	0.913	0.725	2.853
	PU1	0.830				
	PU2	0.900				
	PU3	0.860				
	PU4	0.810				
Satisfaction (SAT)			0.924	0.920	0.743	4.266
	SAT1	0.810				
	SAT2	0.850				
	SAT3	0.880				
	SAT4	0.900				
Trust (TRU)			0.912	0.910	0.717	2.335
	TRU1	0.810				
	TRU2	0.870				
	TRU3	0.920				
	TRU4	0.780				
Continuance Intention (CI)			0.946	0.943	0.806	2.988
	CI1	0.890				
	CI2	0.890				
	CI3	0.910				
	CI4	0.90				

**Table 6:** Construct-level and full measurement model CFA fit results.

Fit Index	Recommended Threshold	CON	CQ	SN	PE	PU	SAT	TRU	CI	Full Measurement Model
CMIN/df	≤3.00	1.103	0.034	1.292	0.656	0.020	0.468	0.438	0.043	2.620
AGFI	≥0.90	0.993	1.000	0.992	0.995	1.000	0.997	0.997	0.997	0.901
GFI	≥0.90	0.999	1.000	0.999	0.999	1.000	1.000	1.000	1.000	0.920
CFI	≥0.90	1.000	1.000	1.000	1.000	1.000	1.000	1.000	1.000	0.969
NFI	≥0.90	1.000	1.000	0.999	1.000	1.000	1.000	1.000	1.000	0.951
TLI	≥0.90	1.000	1.005	0.999	1.001	1.003	1.001	1.001	1.001	0.964
RMSEA	<0.06	0.011	0.000	0.019	0.000	0.000	0.000	0.000	0.000	0.045
SRMR	<0.05	0.002	0.001	0.015	0.007	0.000	0.001	0.002	0.001	0.029

Note: Construct-level fit indices were retained for descriptive purposes, whereas overall model fit was evaluated primarily using the full measurement model CFA.

**Table 7:** Heterotrait–monotrait ratios of correlations.

Construct	CON	CQ	SN	PE	PU	SAT	TRU	CI
CON								
CQ	0.686							
SN	0.607	0.520						
PE	0.641	0.785	0.605					
PU	0.541	0.759	0.415	0.779				
SAT	0.684	0.794	0.564	0.829	0.827			
TRU	0.673	0.623	0.611	0.645	0.542	0.734		
CI	0.586	0.752	0.459	0.763	0.730	0.817	0.687	

**Table 8:** Structural model evaluation using goodness-of-fit indices.

Fit Index	Recommended Threshold	Observed Value	Fit Interpretation
CMIN/df	≤3.00	2.546	Acceptable model fit
AGFI	≥0.90	0.902	Acceptable model fit
GFI	≥0.90	0.922	Acceptable model fit
CFI	≥0.90	0.971	Acceptable model fit
NFI	≥0.90	0.954	Acceptable model fit
TLI	≥0.90	0.966	Acceptable model fit
RMSEA	<0.08	0.044	Acceptable model fit
SRMR	<0.08	0.033	Acceptable model fit

**Table 9:** Results of structural path analysis and hypothesis testing.

Hypothesis	Structural Path	Standardized Estimate	*p*-Value	Statistical Conclusion
H1	Confirmation → Perceived Enjoyment	0.818	***	Significant
H2	Confirmation → Satisfaction	0.244	***	Significant
H3	Content Quality → Satisfaction	0.269	**	Significant
H4	Content Quality → Perceived Usefulness	0.378	***	Significant
H5	Content Quality → Trust	0.601	***	Significant
H6	Subjective Norm → Trust	0.249	***	Significant
H7	Perceived Enjoyment → Satisfaction	0.161	**	Significant
H8	Perceived Enjoyment → Continuance Intention	0.203	***	Significant
H9	Perceived Enjoyment → Perceived Usefulness	0.449	***	Significant
H10	Perceived Usefulness → Satisfaction	0.326	***	Significant
H11	Satisfaction → Continuance Intention	0.559	***	Significant
H12	Trust → Continuance Intention	0.159	***	Significant

*p* < 0.01 = **, *p* < 0.001 = ***.

**Table 10:** Bootstrapped Specific Indirect Effects.

Specific Indirect Path	Standardized Indirect Effect (β)	SE	*p*-Value	95% Bootstrap CI	Result
CON → PE → CI	0.166	0.054	***	[0.060, 0.272]	Supported
CON → PE → SAT → CI	0.074	0.029	***	[0.017, 0.131]	Supported
CON → PE → PU → SAT → CI	0.067	0.019	**	[0.030, 0.104]	Supported
CON → SAT → CI	0.136	0.050	***	[0.039, 0.234]	Supported
CQ → SAT → CI	0.150	0.065	***	[0.022, 0.279]	Supported
CQ → PU → SAT → CI	0.069	0.022	***	[0.026, 0.112]	Supported
CQ → TRU → CI	0.096	0.022	**	[0.053, 0.138]	Supported
SN → TRU → CI	0.040	0.011	***	[0.018, 0.062]	Supported

Note: Confidence intervals were estimated using 5000 bootstrap resamples. Statistical significance was established when the 95% bootstrap confidence interval did not include zero. *p* < 0.01 = **, *p* < 0.001 = ***.

**Table 11:** NCA effect sizes and statistical significance.

Condition	CE-FDH	CR-FDH
Confirmation (CON)	0.037	0.019
Content Quality (CQ)	0.184 ***	0.142 ***
Subjective Norm (SN)	0.021	0.010
Perceived Enjoyment (PE)	0.101 **	0.078 **
Perceived Usefulness (PU)	0.100 **	0.076 **
Satisfaction (SAT)	0.239 ***	0.180 ***
Trust (TRU)	0.042 *	0.021

NCA effect sizes (d). * *p* < 0.05, ** *p* < 0.01, *** *p* < 0.001.

**Table 12:** Bottleneck table based on CE-FDH mean composite scores.

CI Target (%)	CI Score	CON	CQ	SN	PE	PU	SAT	TRU
0	3.000	NN	NN	NN	NN	NN	NN	NN
10	3.400	NN	NN	NN	NN	NN	NN	NN
20	3.800	NN	NN	NN	NN	NN	3.250	NN
30	4.200	2.250	NN	NN	NN	NN	3.250	NN
40	4.600	2.250	NN	NN	NN	NN	3.250	NN
50	5.000	2.250	NN	NN	NN	NN	4.000	NN
60	5.400	2.250	4.000	NN	3.200	NN	4.000	NN
70	5.800	2.250	4.250	NN	3.200	4.250	4.500	NN
80	6.200	2.250	4.500	NN	4.000	4.500	4.500	3.250
90	6.600	2.250	4.500	2.000	4.200	4.500	4.500	3.250
100	7.000	2.250	4.750	2.000	4.200	4.500	4.500	3.250

Note: NN = not necessary at the specified CI target. Bottleneck values were only interpreted substantively for conditions with statistically supported necessity effects.

## Data Availability

The data presented in this study are available within the article. Further inquiries can be directed to the corresponding author.
